# Medical Society of the County of New York

**Published:** 1881-03

**Authors:** 


### MEDICAL SOCIETY OF THE COUNTY OF NEW YORK.

*The Treatment of Extra-Uterine Gestation.*—At a stated meeting of the society held February 28th, Prof. William T. Lusk read a paper on the above subject. In commencing, he alluded to the fact that extra-uterine pregnancy was a condition which existed much more frequently than was formerly supposed to be the case, and stated that for its present knowledge of the subject the profession was greatly indebted, first to the late Dr. John S. Parry, of Philadelphia, and, secondly, to Prof. T. G. Thomas. As cases of this kind were now known to be comparatively common, the question of treatment, therefore, assumed a high degree of importance.

The natural termination of tubal and interstitial pregnancy was by rupture of the sac, hæmorrhage, peritonitis, and death; although there was a certain number of spontaneous recoveries on record, either through the mummification of the fœtus or the formation of a fistulous tract through which it was gradually discharged in small portions. In ovarian and abdominal pregnancies the death of the fœtus might take place either prematurely or at full term, and in the majority of cases it resulted in suppuration, which was almost sure to be followed by peritonitis and the death of the mother. Occasionally, however, the latter survived, and a fistulous opening was established into the vagina or rectum, or through the external abdominal walls, by means of which elimination took place. But this process of elimination was ordinarily a very slow one, and in the greater number of instances the patient required active interference on the part of the medical attendant, or else death would eventually ensue from blood-poisoning or exhaustion. Again, in a certain proportion of cases the fœtus after its death became coated with a bony or earthy crust, and remained as a comparatively innocuous tumor during the rest of the woman's life. Its presence under such circumstances did not prevent the occurrence of pregnancy subsequently.

The treatment of extra-uterine gestation, Dr. Lusk continued, varied according to the stage of pregnancy. In cases of early gestation the indication was plainly to destroy the life of the fœtus, and in order to accomplish this without injury to the mother a number of methods had been suggested and practiced. One of the most simple was the puncture of the sac, and this could usually be easily effected by means of a trocar introduced through the walls of the vagina or rectum. It was true that a number of recoveries were on record in cases where this plan was adopted, but the results, as a rule, were unfavorable. The second method mentioned was that of injecting poisonous solutions into the sac by means of the hypodermic syringe. The first used was one-fifth of a grain of sulphate of atropia in a small quantity of water; but Friedereich had substituted the fifth of a grain of morphia for this, and had reported two successful cases under its use.

The third method was that resorted to by Thomas, namely, the use of the galvano-cautery. One case barely escaped resulting fatally in his hands, however, and in the latest edition of work on diseases of women (which contained a chapter on extra-uterine pregnancy) he recommended Paquelin's cautery. It was to be employed only after the pregnancy had given rise to symptoms of trouble. The fourth and last method considered was the use of the faradic current. It was to be kept up for from five to ten minutes through the seat of the product of conception, and repeated from time to time, if necessary, for one or two weeks, until the shrinkage of the tumor left no doubt that the passage of the current had produced the desired effect. Dr. Lusk then related in detail the history of a very interesting case in which he had called Dr. Thomas in consultation, and in which the faradic current had been employed with complete success. The use of the faradic current, in this connection, he continued, the profession owed to Dr. James G. Allen, of Philadelphia, who reported two cases successfully treated by it in 1872. This method, Dr. Lusk considered, offered a much better chance of recovery than any other, and one great advantage of it was that it was accompanied by no drawbacks. So far as he knew, it had now been resorted to in nine cases, and in every one of them the result had been successful. When it was remembered that in one hundred and fifty cases collected by Hume, there were only seventeen recoveries, the advance that had been made could be better appreciated. The subsequent treatment was that for peritonitis.

The second class of cases was that in which gestation was in advanced stage and the fœtus was still living. Here symptoms of exhaustion finally came on, and the patient was in great danger. Laparotomy in such cases, Dr. Lusk thought, ought to enjoy the highest confidence of the surgeon, provided its performance did not increase the jeopardy of the mother. Parry had reported twenty cases of primary operations, with eight children and six mothers saved; but after a careful study of these he had found that five out of six of the maternal recoveries ought really to be stricken out. The great and unavoidoble danger in such operations was the difficulty or impossibility of removing the placenta.

Finally, he considered the class of cases in which gestation was prolonged after the death of the fœtus. Under these circumstances it was a well established rule not to operate during the continuance of labor pains. The fœtus might at length become converted into a hard, innocuous mass, as mentioned at the outset; but this was the exception, and not the rule. Most commonly it underwent masceration, and the patient sooner or later began to suffer from exhaustion and symptoms of septicæmia. In some cases a fistulous opening was formed into the vagina, into the rectum, through the abdominal walls, or even into the bladder. Under such circumstances the treatment consisted in the enlargement of the fistulous tract, and the extraction of the fœtus piece-meal through it.

Secondary laparotomy was a justifiable operation, and out of thirty-three cases reported there had been nineteen recoveries. Of the two great dangers of the primary operation, hæmorrhage and septicæmia, the first was very greatly diminished by the death of the fœtus and the changes ensuing upon it, and the second could be avoided to a considerable extent by the judicious use of the antiseptic precautions now at the surgeon's command. When circumstances permitted, therefore, it was advisable to delay operative procedure until obliteration of the material vessels had taken place. Whenever the operation was demanded by the condition of the patient, however, antiseptic surgery offered a fair chance of success.

The paper being before the society for discussion, Dr. Jacobi remarked that he had had but one case of extra-uterine pregnancy in his own practice. This occurred some time ago, and he had not treated it by the method so highly recommended by Dr. Lusk, although he was happy to say that the result had been a favorable one. The patient was probably at the beginning of the third month of gestation, and he had punctured the sac through the vaginal walls. He had also made a second puncture two days afterward. Half a year later, when he examined the patient, he had found a hard substance to the left of the uterus which to the touch reminded him of cicatricial tissue in any other part. Even to this day, although it was seventeen years since the operation, the fundus of the uterus was drawn over to the left by means of the contraction that had resulted, while the cervix pointed decidedly toward the right. With his present knowledge of the subject, and especially after listening to the results mentioned by Dr. Lusk in his paper, he would undoubtedly employ the treatment by the faradic current if he were to meet with another similar case.

Dr. Rockwell stated that he had successfully employed electricity in the treatment of three cases. The first was that of Dr. McBurney, which had been alluded to by Dr. Lusk, and which, having been published in full in the *New York Medical Journal*, was well known to the profession at large. The correctness of the diagnosis had been disputed in some quarters, but he did not propose to discuss this point. The second case was that of Dr. Billington, also mentioned in the paper of the evening, and the third he had seen quite recently. Dr. Lusk had spoken particularly of the use of the faradic current, but it seemed to him that the galvanic current would prove the most efficient in destroying the fœtus. In Dr. McBurney's case this was employed, and two applications were made. Before it was resorted to Dr. Thomas, who was one of the consultants, asked him if he could kill the fœtus in this way without doing injury to the mother. The result, as was well known, was entirely satisfactory. In Dr Billington's case he had made but a single application of electricity, and did not know how many had been made subsequently. In the third case there had been but one application—of the galvanic current—altogether, and the recovery had been as successful as in the other instances. These three cases constituted all his experience in regard to this interesting subject, but they at all events went a considerable distance toward settling the point that the fœtus might be destroyed without injury or danger to the mother.

Dr. Billington said that in his case a positive diagnosis was made by Dr. Thomas, whom he called in consultation, and that he had made four applications after the first one by Dr. Rockwell. The first three times he employed fourteen or fifteen cells, but the last time he used the full strength of the battery, thirty-six cells.

Dr. Munde had seen the three cases of extra uterine gestation together. The first was in the service of Scanzoni, and was fatal. It was a case of ventral pregnancy, and the patient went beyond full term. The second occurred in Braun's clinic. The pregnancy had originally been either interstitial or tubal, and the fœtus at length escaped into the abdominal cavity. The woman was in a very cachectic condition, and it was decided to wait till her death before operating for the purpose of saving the child's life. As soon as she expired laparotomy was performed, but the child, which weighed nine pounds, was asphyxiated. The third case occurred in his own practice, and the circumstances were peculiar, the fœtus escaping through the uterine canal, as in Dr. McBurney's case. Before he met with it he had been disposed to doubt the accuracy of the diagnosis in the latter, as he could not understand how the fœtus could pass through the undilated uterine canal. Now, however, he was quite willing to take back all that he had said upon this point, for the case in his own experience had completely convinced him that such a thing was quite possible. In this case he had sounded the uterus with the probe and found it empty, and yet twenty-four hours afterward he found the uterine cavity occupied by the fœtal mass, while the tumor which had previously existed before the uterus had entirely disappeared. In this instance bimanual examination was unusally easy, and there could be no doubt whatever of this latter fact.

It seemed to him that Dr. Rockwell was correct in his view that the galvanic was to be preferred to the faradic current in these cases. There were two reasons that occurred to him why the galvanic was preferable. First, the faradic current had the effect of causing a contraction of the muscular fibres of the tube, and, second, while the faradic current might kill the fœtus by the shock it produced, the galvanic had the additional advantage of decomposing the amniotic fluid.

In Dr. McBurney's case Dr. Emmet had made a suggestion, which he thought might prove of service in a certain proportion of instances. "Why not dilate the uterine cavity, said he, "and extract the fœtus in this way?' This might, perhaps, be a bold and difficult procedure, but, in the hands of an operator as skillful as Dr. Emmet, could no doubt be successfully accomplished in some cases of tubal or interstitial pregnancy. When rupture of the tube had taken place, he believed it to be quite justifiable to p rform laparotomy, as proposed by Kiwisck, and, with all the resources of modern surgery now at one's command, he thought that the prospect of success would be as good as in many bad cases of ovariotomy.

In concluding the discussion, Dr. Lusk remarked that the galvanic current had been proposed in the treatment of extra-uterine pregnancy as early as 1857. At first its introduction in the field of surgery was hailed with great enthusiasm; but afterwards the plan was abandoned because the operator found that they did not obtain as good results from it as from simple puncture of the sac. It was after the use of the galvanic current had been given up that Dr. Allen resorted to the faradic current, and up to the present time (as he had mentioned in the paper) nine cases had been reported in which it had been successfully employed without the occurrence of a single bad symptom. Or, leaving out Dr. McBurney's case, in which he had been under the impression that the faradic current had been used, until corrected by Dr. Rochhel, this evening, there would be eight successful cases on record.

*Proposed Law to Regulate the Sale of Patent and Proprietary Medicines.*—Dr H. O. Piffard presented for the consideration of the society a bill which an effort was now being made to have passed by the legislature, and which he said combined some very objectionable features with some that were really excellent. It was in five sections. The first provided that all patent and proprietary medicines should have their exact composition printed upon the exterior of the bottle, box, or package in which they were sold; the second, that three commissioners, the chairman of whom shall be the president of the county medical society, should have the power of examining all such medicines, and, if necessary, the process of their manufacture, when, if they found nothing injurious in their ingredients, they should be required to furnish a certificate to that effect; the third provided that such commissioners should receive pay at the rate of ten dollars a day for all time actually spent in the performance of their duties; the fourth provided that certain penalties should be inflicted for infringements of the law; and the fifth, that the act should go into effect immediately.

The first and fourth sections, Dr. Piffard said, were altogether unobjectionable and their enforcement, he believed, would be of great service to the profession and the community at large; but the second and third were unnecessary and were open to very serious objections. If the second section were passed by the legislature, all patent medicines that were examined and favorably reported upon by the commissioners would be advertised and offered for sale as endorsed by the medical profession. In regard to the third section he remarked that a work of this kind would take all the time of the commissioners, and that probably no one occupying the position of president of the county medical society would be willing to give up his practice for ten dollars a day. Moreover, as the bill was now constructed, the office of commissioner was apparently for life; no definite time being specified for its continuance. On motion of Dr. Piffard, it was decided that after sections two and three had been stricken out, and one or two slight alterations made in the others, the president and secretary should be authorized to endorse the bill on behalf of the society.—*Boston Medical and Surgical Journal.*

